# Chemical and Biological Properties of Quinochalcone *C*-Glycosides from the Florets of *Carthamus tinctorius*

**DOI:** 10.3390/molecules181215220

**Published:** 2013-12-10

**Authors:** Shijun Yue, Yuping Tang, Shujiao Li, Jin-Ao Duan

**Affiliations:** Jiangsu Collaborative Innovation Center of Chinese Medicinal Resources Industrialization, Nanjing University of Chinese Medicine, Nanjing 210023, China

**Keywords:** *Carthamus tinctorius*, quinochalcone, *C*-glycosides, spectroscopic, biological activity

## Abstract

Quinochalcone *C*-glycosides are regarded as characteristic components that have only been isolated from the florets of *Carthamus tinctorius*. Recently, quinochalcone *C*-glycosides were found to have multiple pharmacological activities, which has attracted the attention of many researchers to explore these compounds. This review aims to summarize quinochalcone *C*-glycosides’ physicochemical properties, chromatographic behavior, spectroscopic characteristics, as well as their biological activities, which will be helpful for further study and development of quinochalcone *C*-glycosides.

## 1. Introduction

*Carthamus* L. is a genus belonging to the tribe *Cynareae* (thistle), subfamily *Tubuliflorae* and family *Compositae*. The eastern part of the Mediterranean region is regarded as the original centre of this genus. The genus includes about 25 species, distributed from Spain and North Africa across the Middle East to northern India [1]. However, *Carthamus tinctorius* L. is the only species of this genus found in China and is believed to have been domesticated somewhere in the Fertile Crescent region over 4,000 years ago [2]. It has been used as a food additive, a natural pigment and a herbal medicine in oriental countries. The historical and popular names of *Carthamus tinctorius* are summarized in Table 1 and full details of flower characteristics data provided in Table 2.

**Table 1:** Historical and popular names of *Carthamus tinctorius* in various languages.

Scientific Language	
Colour Index [3]	Natural Red 26 & Natural Yellow 5
**Semitic and Arabic Languages**	
Aramaic/Hebrew	Qurtami, Qurtema, Qurtam (in modern Hebrew); Dardara & Qotzah (thorn, thistle); Moriqa (of the “thistle”)
Arabic	Osfur, Usfar; Qurtum, Qorton; Khiri
Latin	Carthamus
English	Safflower, Dyer’s thistle, False saffron, Bastard saffron, Dyer’s saffron
**European Languages [4]**	
Italian	Cartamo, Zaffrone, Zaffranone, Zafferano bastardo, Asfore, Grogo
French	Carthame officinal, Faux safran, Graine de perroquet, Safran Bâtard, Safran d’Allemagne, Vermillon de Provence
German	Borstenkraut, Deutscher Saflor, Falscher Saffran, Färber-Saflor, Wilder Saflor, Türkische Saflor
Spanish	Caeramo, Azafaran bastardo, Alazor, Azafran romì
**Asian Languages**	
Chinese [5]	Honghua (red flower), Grass safflower, Huai safflower, Chuan safflower, Du safflower
Japanese [6]	Benibana, Benihana
Hindi [7]	Kusumba, Kusuma, Kusum, Karadai, Hubulkhurtum, Cusumbha, Kamalotarra
Pakistani [7]	Khurtum
Afghan [7]	Muswar, Maswarah, Kajireh, Kariza
Iranian [7]	Kafsha, Kafshe, Kosheh, Zafaran-golu, Kouchan gule, Kah'li, Golbar aftab, Brarta, Kharkhool

**Table 2:** Physical characteristics of *Carthamus tinctorius* during floral development [8].

Days Since Emergence	50	75	100
Stage	Bud formation	Flower formation (petal, stamen, pistil and pollen)	Full flowering
Flower color	Yellow (+++), red (+)	Yellow (++), red (++)	Red (+++), yellow (+)

+: week intensity of red and yellow color; ++: moderate intensity of red and yellow color; +++: high intensity of red and yellow color.

*Carthamus tinctorius* L. is a branching, thistle-like herbaceous annual plant with numerous spines on leaves and bracts [9]. The dried florets of *Carthamus tinctorius* has been used extensively for over 2,500 years in Traditional Chinese Medicine (TCM) to treat stroke, coronary heart disease and angina pectoris [10,11]. In the *Compendium of Materia Medica*, it is described as being able “to invigorate the circulation of blood”, which suggests its potential in circulatory system [12]. *Carthamus tinctorius* is mainly taken as decoction in TCM prescriptions, therefore the water-soluble components should be responsible for the observed therapeutic effects. It is worth mentioning that the water extract of *Carthamus tinctorius* has been developed as an intravenous injection in China and has been extensively applied in hospitals to treat cardiovascular diseases [13].

**Figure 1:**
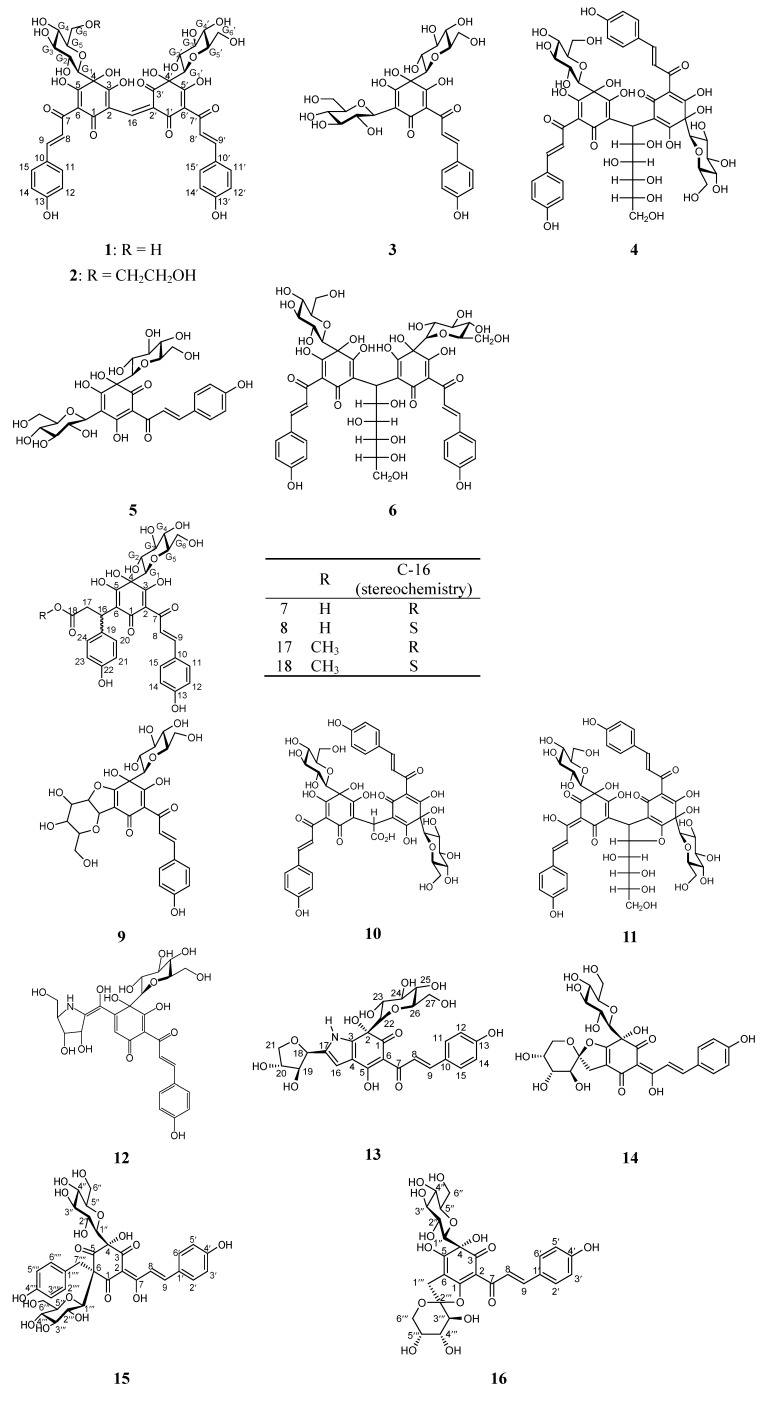
Quinochalcone *C*-glycosides isolated from *Carthamus tinctorius*.

More than 200 compounds have been isolated from *Carthamus tinctorius*, including flavonoids [14,15], alkaloids [16,17], lignans [18,19], carboxylic acids [20], steroids and polysaccharides, where quinochalcone *C*-glycosides, quinone-containing chalcones that are oxidized at the A ring, are regarded as the main active and characteristic compounds in the water extract. Almost all of red and yellow pigments in the petals of *Carthamus tinctorius* are classified as members of the *C*-glucosylquinochalcone family of flavonoids that occurs only in *Carthamus tinctorius* [21]. Up to now, 18 quinochalcone *C*-glycosides have been obtained from *Carthamus tinctorius* (Figure 1). The major red pigment is carthamin (**1**), which is composed of two *C*-glucosylquinochalcone moieties [22]. It was named originally in 1846 by Schlieper, who analysed its chemical structure. Meanwhile, Saito [23] isolated one geometrical isomer of carthamin from a red pigment, and found a marked difference between them via optical rotary dispersion spectroscopy. Unfortunately, there are no further detailed data describing it. Furthermore, a distinct and minor red pigment was isolated from *Carthamus tinctorius*, the structure of which was elucidated as a hydroxyl ether of carthamin named hydroxyethylcarthamin (**2**) by means of NMR and HR-FAB-MA analyses [24]. The major components of yellow pigments are hydroxysafflor yellow A (HSYA, **3**) and safflor yellow B (SYB, **4**) [25]. HSYA, the main active component of safflower yellow (SY), has been demonstrated to restrain the conglomeration of platelet, promote blood circulation, remove blood stasis, have anti-oxidative activity [26], and promote metabolism. Therefore, HSYA is chosen as an active marker component for quality control of safflower in the Chinese Pharmacopoeia [27]. SYB has been extensively used for treatment of cardiocerebrovascular diseases in TCM and has shown a good neuroprotective and antioxidant ability *in vivo* [28]. Besides these two, several other minor quinochalcone *C*-glycosides, including safflomin A (**5**), safflomin B (**6**), safflomin C (**7**), isosafflomin C (**8**), safflor yellow A (SYA, **9**), precarthamin (**10**), and anhydrosafflor yellow B (AHSYB, **11**) have been reported. Among them, Kazuma *et al.* have reported the isolation and structural determination of precarthamin [29,30], which was converted into carthamin both *in vitro* and *in vivo*. In addition, the *N*-containing yellow pigments, tinctormin (**12**) and cartormin (**13**) have also been isolated recently from *Carthamus tinctorius.* Tinctormin was also found to be a potent Ca^2+^ antagonist [31]. Jiang *et al.*, [32,33] have isolated saffloquinoside A (**14**), saffloquinoside B (**15**) and saffloquinoside C (**16**) from the florets of *Carthamus tinctorius*. Saffloquinoside A and saffloquinoside C have uncommon six-five member dioxaspirocycles, while saffloquinoside B has a cyclohexatrione skeleton with a benzyl group and two *C*-glycosyl units. In addition, Methylsafflomin C (**17**) and methylisosafflomin C (**18**) were isolated from the BuOH-soluble fraction of *Carthamus tinctorius* by reversed phase HPLC [34].

During the past 50 years, there has been abundant research focusing on the structural identification of quinochalcone *C*-glycosides. However, quinochalcone *C*-glycosides are very unstable and can easily convert into unknown substances with various hypsochromically modulated colours. Remarkably, Onodera *et al.* [35] took safflomin A and SYA as the same compound in 1981, but they, subsequently, found that safflomin A was unique. It is assumed that these different assignments for safflomin A are partially due to the instability of the compound caused by the C-*β*-d-glucopyranosyl moiety linked to the *α*-carbon between the 1,3-diketone of the characteristic quinochalcone [36]. Besides, temperature, UV-light, pH, gas phase, metal ions, and certain chemicals are all known to be decisive instigators for facilitating colour bleaching [37]. Under UV-C light irradiation, for example, carthamin was more unstable than SYB (average bleaching ratio, carthamin:SYB = 2.7:1.0) [38]. Furthermore, carthamin gradually faded from normal red to reddish orange, orange yellow and finally to pale yellow in aqueous media below pH 6.5. Above pH 7.0, it readily discoloured to a brownish yellow [39]. Thus, there are some structures of quinochalcone *C*-glycosides proposed in preliminary stage of phytochemical researches that have been proved incorrect. As much conflicting data on their structural elucidation have appeared over a period of 50 years in many different sources, it is necessary to figure out the valid structures of quinochalcone *C*-glycosides. Meanwhile quinochalcone *C*-glycosides have a wide range of pharmacological activities, and have become a research hotspot with many scientists committed to synthesizing quinochalcone *C*-glycosides rather than extracting them from *Cathamus tinctorius*. In total synthesis of quinochalcone *C*-glycosides, asymmetric synthesis of carthamin (as the acetate) has been achieved [40], and the stereochemistry of its chiral carbon was determined to be *S* [41]. The total syntheses of the other yellow pigments of quinochalcone *C*-glycosides have not been carried out, but the synthesis of analogs in which the glucosyl group, or the glucosyl and hydroxyl groups on the chiral carbon were replaced by one or two methyl groups has been achieved for safflomin A [42], safflomin C [43], precarthamin [44], and carthamin [45].

Currently, safflower, SY, and HSYA injections are extensively applied in China for the treatment of cardiovascular or cerebrovascular diseases with good clinical effects. However, its major application through intrvenous injection has also contributed to serious adverse reactions such as allergic shock in some cases [46,47]. Meanwhile, Liu *et al.* [48] found that daily intraperitoneal injection of HSYA at a dose of 180 mg/kg for 90 days, but not at a dose of 60 mg/kg, induced a slight nephrotoxicity. Besides, since quinochalcone *C*-glycosides have very poor intestinal membrane permeability resulting in low oral bioavailability, many methods including the addition of an absorption enhancer, chemical modification, and pharmaceutical means of drug delivery have been employed to solve this issue. Ping *et al.* [49,50,51] used Labrafac Lipophile WL 1349 and propylene glycol dicaprylocaprate as oil system in emulsion to enhance the oral bioavailability of water-soluble HSYA. Lv *et al.* [52] have applied a self-double-emulsifying drug delivery system (SDEDDS) consisting of water in oil emulsions and hydrophilic surfactants to improve the absorption of HSYA.

Somewhat surprisingly however, not a single review on this area which deals exclusively with all the physical, chromatographic and spectroscopic data of quinochalcone *C*-glycosides in *Carthamus tinctorius* has appeared yet, hence, this review aims to summarize quinochalcone *C*-glycosides’ melting points, solubility in different solvents, chromatographic behavior, specific optical rotation, UV, IR, MS and NMR data, and X-ray studies correctly, as well as their biological properties, for future research on their structure-activity relationships.

## 2. Physicochemical Properties

### 2.1. Taste and Appearance

Carthamin and hydroxyethylcarthamin appear in the form of red amorphous powders with metallic lusters [24]. Meanwhile, carthamin was recrystallized from dilute acetone to give red metallic lustrous needles [53], but cartormin exhibits yellow needle-like crystals in MeOH [54]. Besides these three, the rest of known quinochalcone *C*-glycosides occur in the form of yellow amorphous powders. All of quinochalcone *C*-glycosides have a slightly bitter taste [55].

### 2.2. Melting Points

The thermal stabilities of quinochalcone *C*-glycosides such as HSYA, SYB, and precarthamin are relatively poor [56]. Therefore, their melting points are relatively low and have been summarized in Table 3.

**Table 3:** Melting points of quinochalcone *C*-glycosides.

Compound	Melting Point (°C)	Reference
Carthamin	>300 (with decomposition); 210.5–212.6	[57,58]
HSYA	184.2; 184–186	[59,60]
Safflomin A	300 (with decomposition)	[35]
Safflomin C	171.3; 300 (with decomposition)	[61,62]
Isosafflomin C	175.0	[61]
SYA	184–187 (with decomposition); 185–188	[59,63]
Cartormin	>230 (with decomposition)	[54]

### 2.3. Solubilities

Yellow quinochalcone *C*-glycoside pigments are soluble in water, dilute alcohol and practically insoluble in anhydrous ethanol, acetone, diethyl ether, petroleum and ethyl acetate. Among them, HSYA is highly soluble in water (its water solubility is about 0.28 mg/mL, 25 °C) but only slightly soluble in oil [64]. Conversely, carthamin is sparingly soluble in water and readily-soluble in alkalis (ammonia solution) [65], but it is unstable in aqueous media below pH 6.5. Carthamin is mainly used in colored chocolate in Japan.

**Figure 2:**
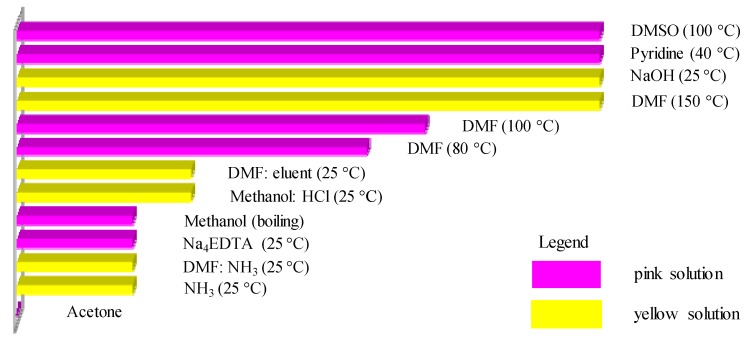
Comparison of the carthamin-extraction abilities of various solvents.

Koren [66] exclusively specialized in carthamin and tested various solvents at various temperatures for their ability to extract carthamin from safflower dyes. The efficiency of extraction was determined by a visual inspection of the color of the textile sample before and after extraction and depth of color of the resulting dye solution. These experimental results are summarized in Figure 2.

### 2.4. Partition Coefficient (K)

The *K* values of HSYA in different two-phase solvent systems have been reported (see Table 4) [67].

**Table 4:** *K* values of HSYA in the different two-phase solvent systems.

No.	Solvent System	Ratio (v/v)	*K*
1	*n*-Butanol-water	1:1	0.03
2	*n*-Butanol-acetone-water	4:1:5	0.14
3	*n*-Butanol-methanol-water	5:1:5	0.06
4	*n*-Butanol-methanol-water	6:1:4	0.30
5	*n*-Butanol-0.1 mol/L HCl	1:1	1.15

## 3. Chromatographic Behavior

### 3.1. Thin-Layer Chromatography (TLC) on Silica Gel

TLC is a very useful analytical tool widely used in Traditional Chinese Medicine because of its simplicity, relatively low cost, high sensitivity, and speed of separation. As the main active and characteristic ingredients in *Carthamus tinctorius*, quinochalcone *C*-glycosides have been chosen as evaluation index for TLC in the Chinese Pharmacopoeia. The TLC of *Carthamus tinctorius* from different geographical regions eluted with *n*-butanol-glacial acetic acid-H_2_O (4:1:2) and detected at 254 nm, showed a very homogeneous pattern of yellow and red pigments with carthamin as a red pigment at *R_f_* = 0.52 and several yellow pigments (e.g., HSYA) between *R_f_* = 0.32 and 0.49 [68].

Fatahi *et al.* [69] identified carthamin and SY by a TLC method with distilled water-isobutanol-ethanol-formic acid (4:7:4:4) as eluent system. The *R_f_* values are given in Table 5. In both types of gels, carthamin ascended in the form of a red horizontal line, but SY ascended in the form of a circular yellow spot for silica gel G and a tailed spot for silica gel Kieselgel 60 F_254_ (1005554 Merck). Precarthamin was very hygroscopic and gave a yellow single spot by TLC [70]. Besides, safflomin A was confirmed to be unstable on silica-gel TLC by Sato *et al.* [39], and its structure is altered in the presence of a small amount of trifluoroacetic acid in methanol at room temperature after 1 day. The *R_f_* values of the different quinochalcone *C*-glycosides have been summarized in Table 5.

**Table 5:** The *R_f_* values of quinochalcone *C*-glycosides.

Sample	Silica Gel Type	Developing Solvent	*R_f_* Values	Reference
Carthamin	Silica gel G (type 60)	*n* -Butanol-glacial acetic acid-H_2_O (4:1:2)	0.93	[69]
	Kieselgel 60 F_254_	*n* -Butanol-glacial acetic acid-H_2_O (4:1:2)	0.88	[69]
	-	*n* -BuOH-AcOH-H_2_O (4:1:5, upper layer)	0.399	[71]
	-	*n* BuOH-EtOH-H_2_O (4:1:2)	0.573	[71]
	-	*n* -BuOH-toluene-pyridine-H_2_O (5:1:3:3)	0.679	[71]
	Merck Kieselgel 60 F_254_	*n* -BuOH-HOAc-H_2_O (4:1:2)	0.49	[25]
SY	Silica gel G (type 60)	*n* -Butanol-glacial acetic acid-H_2_O (4:1:2)	0.85	[69]
	Kieselgel 60 F_254_	*n* -Butanol-glacial acetic acid-H_2_O (4:1:2)	0.78	[69]
HSYA	Merck Kieselgel 60 F_254_	*n* -BuOH-HOAc-H_2_O (4:1:2)	0.33	[25]	
	-	*n* -BuOH-EtOH-H_2_O (4:1:2)	0.34	[49]	
SYB	Merck Kieselgel 60 F_254_	*n* -BuOH-HOAc-H_2_O (4:1:2)	0.42	[25]	
	-	*n* -BuOH-EtOH-H_2_O (4:1:2)	0.42	[56]	
Precarthamin	-	*n* -BuOH-EtOH-H_2_O (4:1:2)	0.46	[56]	
Safflomin C	Merck Kieselgel 60 F_254_	EtOAc-MeOH-H_2_O (100:16:12)	0.2	[25]	
Tinctormin	Merck Kieselgel 60 F_254_	EtOAc-MeOH-H_2_O (100:16:12)	0.15	[25]	
Cartormin	-	*n* -BuOH-EtOH-H_2_O (4:1:2)	0.62	[72]	

### 3.2. Reversed-Phase HPLC (RP-HPLC)

Reversed-phase HPLC has been widely applied for the separation, purification, content determination, and pharmacokinetics of quinochalcone *C*-glycosides. A rapid separation of safflomin A, SYB, SYA, and safflomin C took place on a 150 × 4.6 mm reversed-phase KR1005 C_18_ (Kromasil & reg) column at room temperature and was completed within 20 min in combination with the solvent systems: mobile phase A ‒ ultra-pure water/trifluoroacetic acid (99.8/0.2, V/V); and mobile phase B ‒ acetonitrile/trifluoroacetic acid (99.8/0.2, V/V) at 1.0 mL/min [73,74]. Yao *et al.* [75] established an RP-HPLC method for simultaneously determination of HSYA and SYA in *Carthamus tincotrius*. The analytical column was Akasil C18 (4.6 mm × 150 mm, 5 μm), and the mobile phase was composed of acetonitrile and 0.4% phosphoric acid at a flow rate of 0.7 mL/min. Besides, when methanol-formic acid in water (0.2%) and acetonitrile were used as mobile phase (H_2_O-acetonitrile-MeOH = 62:2:36) on ODS C_18_ (250 mm × 4.6 mm I.D., 5 μm) in isocratic mode, HSYA showed a good separation from other major peaks [59].

A simple and reproducible RP-HPLC method for quantification of HSYA in rat plasma and tissues after oral administration of safflower extract or SY was developed by Li *et al.* [76]. The HPLC determinations were achieved using a Hypersil BDS-C_18_ column (250 × 4.6 mm, 5 μm) and an Eclipse XDB-C18 guard column (12.5 × 4.6 mm, 5 μm). The mobile phase consisted of acetonitrile and 0.3% aqueous acetic acid at a flow rate of 1.0 mL/min.

Yang *et al.* [77,78,79] have investigated the pharmacokinetics of HSYA in healthy Chinese female volunteers, patients with blood stasis syndrome, normal and acute blood stasis rats. The analysis of HSYA in plasma was performed on a Shimadzu liquid chromatographic system. The analytical column employed was a Shim-pack VP-ODS C18 column (150 mm × 4.6 mm I.D., 5 μm particle size) protected with a ODS guard column (10 mm × 4.6 mm I.D., 5 μm particle size). The mobile phase was composed of acetonitrile and 0.022 M KH_2_PO_4_ (adjusted to pH 3.5 with orthophosphoric acid) at 0.8 mL/min.

### 3.3. Capillary Zone Electrophoresis (CZE)

Capillary electrophoresis has been used as an attractive method for separating and monitoring TCMs owing to its high resolving power, low solvent consumption, and simple pretreatment [80]. Since yellow pigments of quinochalcone *C*-glycosides have several diol groups, CZE was performed with a borate buffer at various alkaline pH values. Good separation was obtained at pH 9.0, in spite of the long time to complete the separation. However, micellar electrokinetic chromatography (MEKC) with 2.0% butyl acrylate/butyl methacrylate/methacrylic acid copolymer sodium salts (BBMA) in an ammonium formate buffer at pH 7.0 gave better resolution and shorter analysis time of yellow pigments. Red pigments were also successfully separated by MEKC with BBMA solution [81].

Jiang *et al.* [82] further developed a new CZE method for simultaneous assay of four quinochalcone *C*-glycosides, HSYA, SYA, safflomin C, and safflomin A in the Chinese herbal extract of *Carthamus tinctorius* L. The optimum buffer system was 30 mM borate buffer (Na_2_B_4_O_7_/HCl, pH 9.00) with 10% (v/v) methanol. The voltage was 15 kV and detection was at 270 nm. Regression equations revealed linear relationships (correlation coefficients: 0.9973, 0.9992, 0.9989, and 0.9996) between the peak of each compound and its concentration. The within-day relative standard deviations of migration times and peak areas were <1.53 and 4.14%, respectively. The contents of four quinochalcone *C*-glycosides were successfully determined with satisfactory repeatability and recovery.

### 3.4. High-Speed Countercurrent Chromatography (HSCCC)

The stationary phase of HSCCC is liquid instead of solid compared with other chromatographic techniques. Advanced centrifugal partition technology is used to hold the liquid stationary phase in the colum, which is beneficial to separate natural compounds. Inoue *et al.* [83] separated and purified safflomin A and safflomin B from Carthamus yellow using a two-phase solvent composed of tert-butyl methyl ether/*n*-butanol/acetonitrile/0.5% aqueous trifluoroacetic acid solution (2/2/1/5, V/V) within 6 h. HSCCC was performed using an HSCCC-1A prototype model (multi-layer coil planet centrifuge, Shimadzu Co., Kyoto, Japan) with a 10 cm orbital radius that produces a synchronous type-J planetary motion at maximum speed of 800 rpm. The multi-layer coil was prepared by winding a *ca.* 160 m length of PTFE tubing onto the column holder with a 10 cm hub diameter and a 15 cm hub length, making six coiled layers with a total capacity of 270 mL.

## 4. Spectroscopic Characteristics

### 4.1. Specific Optical Rotation

In Table 6, the specific optical rotations of reported quinochalcone *C*-glycosides are provided as follows.

**Table 6:** Specific optical rotation of quinochalcone *C*-glycosides.

Compound	[α]_D_ (°)	Solvent	Concentration (g/100 mL)	Reference
Carthamin	−57.3	Me_2_CO	20	[84]
HSYA	−54.3	MeOH	10	[25]
SYB	+208	MeOH	10	[25]
Safflomin C	−99.1	MeOH	10	[61]
Isosafflomin C	−114.7	MeOH	10	[61]
SYA	−164.5	MeOH	6	[85]
Tinctormin	−206	MeOH	10	[31]
Cartormin	−153.4	Pyridine	1.23	[86]
Saffloquinoside A	−24.6	MeOH	4	[32]
Saffloquinoside B	−215	MeOH	7	[32]
Saffloquinoside C	−30.6	MeOH	6	[33]
Methylsafflomin C	+22.4	MeOH	3	[34]
Methylisosafflomin C	−16.0	MeOH	3	[34]

### 4.2. Ultraviolet Visible (UV-Vis) Spectra

UV-vis spectra data of quinochalcone *C*-glycosides were provided in Table 7. The most prominent factor in quinochalcone *C*-glycosides is that the band I in the UV-vis spectra is around 403 nm, which allows them to be differentiated from other glycoside flavonoids [70]. Since yellow pigments of quinochalcone *C*-glycosides have similar UV-vis absorption wavelengths, UV-vis spectrophotometry have been applied to determine the total content of SY other than single component. Guo *et al.* [87] analysed the total content of SY by UV at 403 nm. The content of SY was 24.9%–40.34% in *Carthamus tinctorius* from different sources.

**Table 7:** UV-vis spectra data of quinochalcone *C*-glycosides.

Compound	Solvent	l_max_ Value (log e) (nm)	Reference
Carthamin	EtOH	515 (4.69), 377 (4.28), 244 (4.13)	[21]
HSYA	MeOH	403 (4.51), 226 (4.30); 399 (4.00)	[21,56]
SYB	MeOH	410 (4.77), 239 (4.43); 410 (4.55)	[21,56]
Safflomin C	MeOH	406 (4.37), 346 (sh), 230 (4.26)	[61]
Isosafflomin C	MeOH	407 (4.53), 348 (sh), 230 (4.44)	[61]
SYA	MeOH	400, 334 (sh), 224	[63]
Precarthamin	MeOH	406 (4.66), 238 (4.36); 417 (4.47)	[21,56]
	EtOH	423 (4.56), 343 (4.25)	[88]
AHSYB	MeOH	410 (4.62), 230 (4.33)	[21]
Tinctormin	MeOH	405 (4.5), 275 (4.5)	[25]
Cartormin	MeOH	406 (4.03), 245 (sh), 221 (3.78)	[72]
Saffloquinoside A	MeOH	404 (4.25), 314 (3.54), 243 (3.86), 202 (3.66)	[32]
Saffloquinoside B	MeOH	389 (3.64), 282 (3.11), 222 (3.43), 205 (3.55)	[32]
Saffloquinoside C	MeOH	428, 348, 263	[33]
Methylsafflomin C	MeOH	404, 340 (sh), 227	[34]
Methylisosafflomin C	MeOH	408, 337 (sh), 231	[34]

### 4.3. Infrared (IR) Spectra

IR spectra data of quinochalcone *C*-glycosides is listed in Table 8. In particular, the IR spectrum of cartormin revealed the presence of a keto-enol system (1600–1640 cm^−1^) and hydroxyl groups which were due to sugar moieties (3400, 1070 (br) cm^−1^), and the n_C-N_ absorption was found at 1269 cm^−1^ [88].

**Table 8:** IR spectra data of quinochalcone *C*-glycosides.

Compound	Matrix	Wave Numbers (cm^−1^)	Reference
Carthamin	KBr	3370, 1740, 1675, 1622, 1600, 1584, 1512	[21]
HSYA	KBr	3381, 1676, 1622, 1601, 1516	[21]
SYB	KBr	3388, 1680, 1623, 1600, 1517	[21]
Safflomin C	KBr	3365, 1669, 1600; 3400, 1700, 1613, 1595, 1510, 1400, 1230, 1162, 1068, 920, 825	[62,68]
Isosafflomin C	KBr	3354, 1670, 1600	[62]
SYA	KBr	3380, 1650, 1620, 1600, 1515, 1505, 1170, 1075, 1025, 930	[65]
Precarthamin	KBr	3352, 1669, 1621, 1599, 1583, 1521	[21]
AHSYB	KBr	3188, 1653, 1620, 1600, 1559	[21]
Tinctormin	KBr	3400, 1620, 1600	[25]
Cartormin	KBr	3400, 1640, 1600, 1269, 1070	[86]
Saffloquinoside A	KBr	3381, 2935, 1625, 1598, 1521, 1439, 1252, 1171, 1088, 964, 918, 832, 727	[32]
Saffloquinoside B	KBr	3383, 2932, 1726, 1668, 1616, 1583, 1516, 1441, 1405, 1248, 1171, 1088, 932, 906, 834	[32]
Saffloquinoside C	KBr	3368, 2928, 1651, 1606, 1511, 1369, 1208, 1103, 1089, 1001, 969	[33]

### 4.4. Mass Spectrometry (MS)

**Figure 3:**
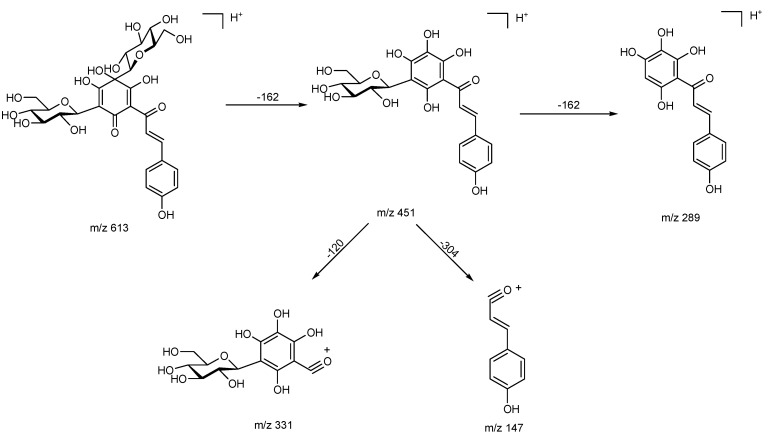
Common fragmentations proposed for quinochalcone *C*-glycosides in positive ion mode.

During the past few years, LC-MS has been frequently employed for analysis of quinochalcone *C*-glycosides, mostly for quantitative purposes. Jin *et al.* [89] characterized quinochalcone *C*-glycosides by UPLC/Q-TOFMS. Their fragmentation showed a special cleavage at the C-C bond except for the typical internal cleavage at the sugar moiety of other *C*-glycosyl flavonoids. In positive ion mode, cleavage of the 5'-glucose produced an [M+H−162]^+^ ion by a neutral loss, which is elucidated in Figure 3 (HSYA was taken as an example), while cleavage of the 5'-glucose in negative ion mode led to an [M−H−163]^-^^·^ ion by radical cleavage. The common fragmentation pathways for quinochalcone *C*-glycosides in negative ion mode were summarized in Table 9. Besides, the cleavage from the carbonyl group produced fragment ions of quinochalcone *C*-glycosides in both ion modes, and fragment ions containing the B ring were used to judge the different substituent groups at the 3'-position.

**Figure 4:**
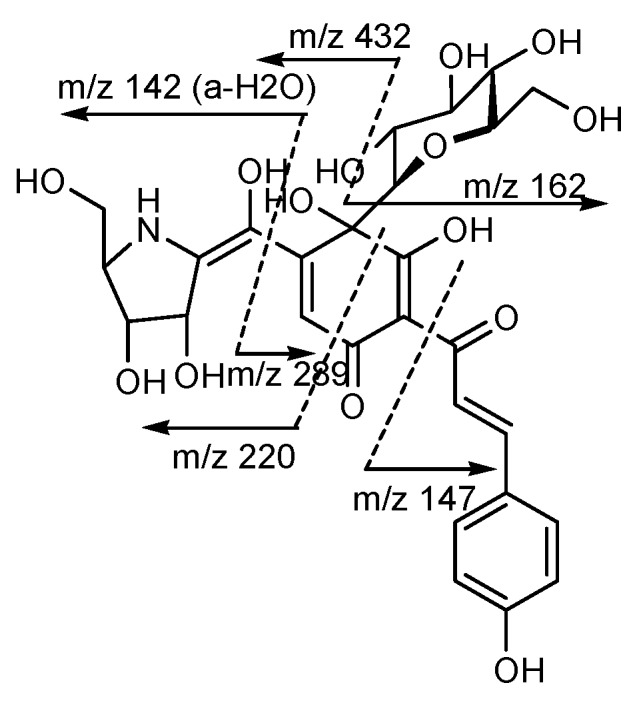
The linked scan FAB-MS of tinctormin in positive ion mode [25].

The structure of carthamin, HSYA, SYA, AHSYB and SYB were analyzed and identified by electro-spray ionization multistage mass spectrometry (ESI-MS^n^) in the negative mode, which mainly produced [M-H]^-^ ion peak in negative ion mode [90,91]. Besides, the linked scan FAB-MS (positive ion mode) of tinctormin was displayed in Figure 4.

**Table 9:** The common fragmentations of quinochalcone *C*-glycosides in negative ion mode.

Attribution of Fragment Ions	Fragmentation Pathway (*m/z*)			
	HSYA	SYA	Safflomin C	Cartormin
[M−H]^-^^·^	611	593	613	574
[M−H−163]^-^^·^	611→448	593→430	613→450	574→411
Fragmentation 1		593→473	613→493	574→454
			613→551→431	
Fragmentation 2		593→447	613→467	574→428
			613→551→405	

### 4.5. NMR Data

NMR data of quinochalcone *C*-glycosides have been published by many different authors. The most complete or reliable studies on reported quinochalcone *C*-glycosides were summarized in Table 10, Table 11, Table 12, Table 13, Table 14 and Table 15. Due to the keto-enol systems in quinochalcone *C*-glycosides, they can easily form an internal hydrogen bond, such as cartormin with an unusual downfield chemical shift of OH-5 (*δ*_H_: 17.87, 1H, s). NMR data of cartormin are summarized in Table 10.

**Table 10:** NMR for cartormin (400 MHz, in DMSO-*d_6_*) [86].

Position	^1^H	^13^C	HMBC
1		196.2	OH-2
2		78.3	OH-2
3		142.2	OH-2, H-16
4		114.8	H-16, -NH-
5		185.7	
6		109.3	
7		180.4	H-8, H-9
8	7.36 (d, *J* = 15.8 Hz, 1H)	118.8	
9	7.65 (d, *J* = 15.8 Hz, 1H)	141.3	H-11, H-15
10		126.3	H-8, H-9, H-12, H-14
11	7.56 (d, *J* = 8.4 Hz, 2H)	130.6	H-8, H-9, H-15
12	6.84 (d, *J* = 8.4 Hz, 2H)	116.0	H-14
13		160.0	H-11, H-12, H-14, H-15
14	6.84 (d, *J* = 8.4 Hz, 1H)	116.0	H-12
15	7.56 (d, *J* = 8.4 Hz, 1H)	130.6	H-8, H-9, H-11
16	6.37 (s)	103.4	H-18, -NH-
17		135.0	H-16, -NH-
18	4.53 (d, *J* = 7.6 Hz, 1H)	76.6	H-21
19	4.06 (m)	76.0	H-18, H-21, OH-19
20	4.11 (m)	70.5	H-21
21	3.62 (m)4.13 (m)	72.9	
22	3.29 (d, *J* = 9.5 Hz, 1H)	84.2	OH-2, H-23, OH-23
23	3.43 (overlap)	69.1	H-22, H-24
24	3.12 (m)	78.5	H-23, H-25
25	3.11 (m)	69.3	H-24
26	2.86 (m)	79.5	H-22
27	3.50 (overlap)	60.7	OH-27

**Table 11:** .^13^C-NMR data of the (E)-olefinic carbon of quinochalcone *C*-glycosides [7].

	Compuond	δ_C_ C-8	δ_C_ C-9	Solvent	Reference
1-enol-3,7-diketo	HSYA	122.8	135.9	DMSO-*d_6_*	[6]
	Safflomin C	123.2	135.1	DMSO-*d_6_*	[6]
	HSYA	124.2	138.1	Pyridine-*d_5_*	[6]
	Safflomin C	122.1	139.4	Pyridine-*d_5_*	[32]
7-enol-1,3-diketo	Saffloquinoside A	117.9	142.4	DMSO-*d_6_*	[18]
	Saffloquiniside B	118.3	143.5	DMSO-*d_6_*	[18]
	SYA	118.0	143.0	DMSO-*d_6_*	[6]
	Cartormin	118.8	141.3	DMSO-*d_6_*	[6]

By NMR techniques, particularly HMBC experiments, Feng *et al.* [7] found that the structure of HSYA in various solutions existed as a dynamic mixture of tautomers, and the major tautomer in the different solvents was the 1-enol-3,7-diketo form. They also found that the ^13^C-NMR data of the (*E*)-olefinic carbon of quinochalcone *C*-glycosides followed a specific trend, which was useful in the identification of the position of the hydroxyl and carbonyl groups in these types of compounds. For instance, in DMSO-*d_6_*, the olefinic carbon signals of the 1-enol-3,7-diketo form occurred at approximately *δ* 123.0 (C-8) and 135.0 (C-9), but the carbons of the 7-enol-1,3-diketo form resonated at approximately *δ* 118.0 (C-8) and 142.0 (C-9). The spectroscopic data of the quinochalcone *C*-glycosides in some solvents are summarized as follow in Table 11.

**Table 12:** ^13^C-NMR and HMBC data of safflomin C and isosafflomin C (100 MHz, in CD_3_OD) [61].

Carbon	Safflomin C		Isosafflomin C	
	^13^C	HMBC (C→H)	^13^C	HMBC (C→H)
1	192.28	16	192.11	16
2	108.39		108.60	
3	195.54		195.59	
4	82.12	G1, G2	82.12	
5	173.20	16	172.89	16
6	113.84	16, 17	113.86	16, 17
7	180.36	8, 9	180.39	8, 9
8	119.25	9	119.25	
9	143.34	11, 15	143.31	11, 15
10	128.22	8, 9, 12, 14	128.22	8, 9, 12, 14
11, 15	131.49	9, 12, 14	131.49	9
12, 14	116.68	11, 15	116.67	11, 15
13	161.07	11, 12, 14, 15	161.09	11, 12, 14, 15
16	36.65	17, 20, 24	36.19	17, 20, 24
17	38.72	16	37.56	16
18	176.62	16, 17	176.48	16, 17
19	135.52	16, 17, 21, 23	135.53	16, 17, 21, 23
20, 24	129.45	16, 21, 23	129.51	16
21, 23	115.49	20, 24	115.58	20, 24
22	156.29	20, 21, 23, 24	156.32	20, 21, 23, 24
G1	88.23	G2	88.23	G2
G2	70.44	G1, G3	70.45	G1, G3
G3	79.57	G2, G4	79.59	G2, G4
G4	69.75	G3, G6	69.68	G3, G6
G5	80.63	G4, G6	80.57	G4
G6	61.32	G4	61.30	G4

The ^13^C-NMR data of safflomin C and isosafflomin C are essentially the same except for the values at C-17 and C-16 (see Table 12). C-18 as the carbonyl carbon of carboxylic acid exhibited high *δ* value (*δ*_C_: 176.62 and 176.48 ppm, respectively). Fewer studies have been carried out on methylsafflomin C and methylisosafflomin C. The NMR data of methylsafflomin C were reported as follows: ^1^H-NMR (400 MHz, CD_3_OD) δ: 7.71 (1H, d, *J* = 15.6 Hz, H-9), 7.50 (2H, d, *J* = 8.4 Hz, H-11, 15), 7.43 (1H, d, *J* = 15.6 Hz, H-8), 7.19 (2H, d, *J* = 8.4 Hz, H-20, 24), 6.79 (2H, d, *J* = 8.4 Hz, H-12, 14), 6.64 (2H, d, *J* = 8.4 Hz, H-21, 23), 4.71 (1H, dd, *J* = 8.0, 8.0 Hz, H-16), 3.70 (1H, br d, *J* = 12.0 Hz H-G6), 3.58 (3H, s, OMe), 3.51 (1H, dd, *J* = 12.0, 2.0 Hz H-G6), 3.47 (1H, d, overlapped, H-G1), 3.46 (1H, overlapped, H-G2), 3.39 (1H, dd, *J* = 9.2, 9.2 Hz, H-G4), 3.22 (1H, dd, *J* = 9.2, 9.2 Hz, H-G3), 3.21 (1H, overlapped, H-17), 3.09 (1H, dd, *J* = 16.0, 8.0 Hz, H-17), 2.92 (1H, br d, *J* = 9.2 Hz, H-G5); ^13^C-NMR (100 MHz, CD_3_OD) δ: 195.50 (C-3), 192.53 (C-1), 180.22 (C-7), 174.98 (C-18), 172.50 (C-5), 161.14 (C-13), 156.39 (C-22), 143.41 (C-9), 135.43 (C-19), 131.51 (C-11, 15), 129.47 (C-20, 24), 128.22 (C-10), 119.14 (C-8), 116.68 (C-12, 14), 115.49 (C-21, 23), 112.50 (C-6), 108.42 (C-2), 88.19 (C-G1), 80.54 (C-G5), 78.41 (C-G3), 78.41 (C-4), 70.39 (C-G2), 69.54 (C-G4), 61.02 (C-G6), 51.98 (C-OMe), 38.57 (C-17), 36.51 (C-16). Only the ^1^H-NMR of methylisosafflomin C has been published: ^1^H-NMR (400 MHz, CD_3_OD) δ 7.70 (1H, d, *J* = 16.0 Hz, H-9), 7.49 (2H, d, *J* = 8.4 Hz, H-11, 15), 7.42 (1H, d, *J* = 16.0 Hz, H-8), 7.16 (2H, d, *J* = 8.8 Hz, H-20, 24), 6.78 (2H, d, *J* = 8.4 Hz, H-12, 14), 6.63 (2H, d, *J* = 8.8 Hz, H-21, 23), 4.77 (1H, dd, *J* = 9.2, 7.2 Hz, H-16), 3.77 (1H, br d, *J* = 11.6 Hz, H-G6), 3.69 (1H, dd, *J* = 11.6, 2.4 Hz, H-G6), 3.59 (3H, s, OMe), 3.52 (1H, overlapped, H-G1), 3.52 (1H, overlapped, H-G2), 3.41 (1H, dd, *J* = 9.6, 9.6 Hz, H-G4), 3.26 (1H, overlapped, H-G3), 3.25 (1H, overlapped, H-17), 3.08 (1H, dd, *J* = 15.6, 7.2 Hz, H-17), 3.05 (1H, m, H-G5) [34]. Compared with safflomin C and isosafflomin C, the *δ*_C-18_ of methylsafflomin C was 174.98 ppm, which suggested the shielding effect of the carbonyl carbon in carboxylic acid derivatives was stronger than that in carboxylic acid.

The ^13^C-NMR and the ^1^H-NMR spectrum of hydroxyethylcarthamin indicated it has the same skeleton as that of carthamin, however, hydroxyethylcarthamin has an additional hydroxyethyl group on one quinochalcone *C*-glycoside moiety according to the HMQC, DEPT and TOCSY experiments, while a correlation was observed between δ3.68 (E1) and δ66.6 (G6') with HMBC experiments. In the ^13^C-NMR spectra, there are two unpaired peaks (*δ* 35.94 and *δ* 176.31 ppm) in precarthamin and carthamin. In the ^1^H-NMR spectra, similar signals of precarthamin to those of carthamin, except for the single peak at *δ* 7.03 (1H), were observed. The signal at d 35.94 assigned to a methine carbon in the DEPT spectrum showed a correlated peak with the proton at *δ* 7.03 in the ^13^C-^1^H COSY spectrum. The other signal at *δ* 176.31 was assigned to a carboxyl carbon because of the chemical shift value. But H-16 of carthamin as alkenyl hydrogen possessed higher chemical shift value at d 9.43 than that of precarthamin as methine hydrogen at *δ* 7.03.

The ^1^H- and ^13^C-NMR data for AHSYB and SYB are summarized in Table 13. It suggests AHSYB possesses the same carbon skeleton as SYB. In the ^1^H-NMR spectra, significant differences between the two were observed in the chemical shifs of G"2 (*∆δ* 1.65) and in the coupling constants at G"1, -2, -3, and -4 of the 1-deoxyglucitol moiety. In the ^13^C-NMR spectra, the signals of six oxygen-substituted sp^2^ carbon atoms in the two cyclohexadienone rings were observed from 171.14 to 202.87 ppm in anhydrosafflor yellow B, and from 175.47 to 197.22 ppm in SYB.

In the ^1^H-NMR spectra, a very characteristic downfield proton signal at *δ* 17.42 (1H, br s) in saffloquinoside A was assignable to an enolic hydroxyl owing to forming an internal hydrogen bond. In the ^13^C-NMR spectra, *δ*_C-1_ and *δ*_C-3_ were 187.5 ppm and 193.8 ppm, respectively, which indicated that C-1 and C-3 were carbonyl carbons and the quinocycle unit in saffloquinoside A should be the cyclohexaendione moiety, instead of a cyclohexadienone as in HSYA. In consideration of the data in Table 14, the NMR data of saffloquinoside C were similar to those of saffloquinoside A. However, several chemical shifts including C-1 (upfield, *∆δ*_C_ -10.6 ppm) and C-5 (downfield, *∆δ*_C_ +13.7 ppm) in quinocycle unit of saffloquinoside C were changed. Furthermore, in the HMBC experiment, the correlations of H-1'" α (*δ*_H_ 2.96) and H-1'" β (*δ*_H_ 2.35) with C-5 (*δ*_C_ 186.8) and C-1 (*δ*_C_ 176.9) in saffloquinoside C revealed that the fructopyranose moiety should be directly linked to C-6 of the quinocycle unit. Thus, saffloquinoside C was a rare quinchalcone glycoside with an uncommon member dioxaspirocycle fused at C-1 and C-6. Besides, saffloquinoside B presented six carbon signals of a phenyl moiety at δ 125.0 (C-1''''), 130.8 (C-2'''', C-6''''), 114.7 (C-3'''', C-5''''), 156.1 (C-4''''), and a methylene signal at δ 43.5 (C-7'''') in the ^13^C-NMR spectrum, which didn’t exist in saffloquinoside A and saffloquinoside B.

The ^1^H- and ^13^C-NMR data in Table 15 suggest that tinctormin has a pattern similar to that of HSYA and SYA, but the significant difference among these three was observed in the chemical shifts of C-1". The value of *δ*_C-1__"_ in tinctormin is 140.9 ppm, indicating that C-1" exists in the enol form rather than the keto form to establish the enamine system with the NH-group.

**Table 13:** NMR data for precarthamin, carthamin, hydroxyethylcarthamin, AHSYB, and SYB.

Compound	Precarthamin		Carthamin		Hydroxyethylcarthamin		AHYB		SYB	
	^1^H ^a)^	^13^C ^a)^	^1^H ^b)^	^13^C ^b)^	^1^H ^b)^	^13^C ^b)^	^1^H ^b)^	^13^C ^b)^	^1^H ^b)^	^13^C ^b)^
MHz	400	100	800	150	800	150	400	100	400	100
1, 1'		193.69, 193.58		188.4		188.0		202.87, 196.99		197.22, 196.51
2, 2'		107.02, 106. 67		113.1		114.0		104.24, 112.42		103.67, 113.92
3, 3'		174.04, 173.47		192.5		193.3		189.31, 187.94		190.66, 188.77
4, 4'		81.46, 81.29		89.6		89.3		85.32, 87.44		79.43, 86.05
5, 5'		172.66		194.2		194.3		186.84, 171.14		184.64, 175.47
6, 6'		107.74		109.5		110.2		103.74, 108.42		107.38, 109.39
7, 7'		178.64, 178.56		183.3		182.2		180.08, 171.31		181.15, 180.94
8, 8'	7.30 (*d*, 16)	118.91	8.21 (*d*,*15.8*)	121.2	8.26 (*d*,*15.6*)	121.1	8.24 (d, 16.0)	122.52	8.39 (d, 15.6)	123.40
	7.28 (*d*,*16*)						8.28 (d, 16.0)	119.20	8.20 (d, 15.6)	119.88
9, 9'	7.58 (*d*,*16*)	140.60,	8.03 (*d*,*15.8*)	141.5	8.11 (*d*,*15.6*)	141.7	7.96 (d, 16.0)	138.89	8.00 (d, 15.6)	138.25
		140.40					7.79 (d, 16.0)	141.54	7.87 (d, 15.6)	142.12
10		126.35		127.4		127.3		127.67		127.28
11, 11', 15, 15'	7.52 (*d*,*8.8*)	130.45	7.50 (*d*,*7.6*)	130.7	7.52 (*d*,*8.5*)	130.9	7.33 (d, 8.4)	130.30	7.52 (d, 8.4)	130.28
	7.50 (*d*,*8.8*)						7.80 (d, 8.4)	131.40	7.35 (d, 8.8)	130.88
12, 12', 14, 14'	6.83 (*d*,*8.8*)	115.93	6.89 (*d*,*7.6*)	116.3	6.87 (*d*,*8.5*)	116.4	6.81 (d, 8.4)	116.35	6.90 (d,8.4)	116.47
	6.80 (*d*,*8.8*)						7.18 (d, 8.4)	116.83	6.88 (d, 8.8)	116.53
13, 13'		159.83, 159.78		160.6		161.0		160.17, 161.29		160.17, 161.06
16	4.82 (*s*)	36.55	9.31 (*s*)	143.9						
17		189.81								
G1, G1'	3.50 (*d*,*9.6*)	86.84,	4.90 (*d*,*9.4*)	86.3	4.99 (*d*,*9.4*)	87.0	4.96 (d, 9.6)	86.98	4.63 (d, 9.6)	85.39
		86.46					4.55 (d, 9.6)	88.13	4.72 (d, 9.6)	88.20
G2, G2'	3.33 (*m*)	68.87	4.45 (*t*,*9.4*)	70.9	4.59 (*t*,*9.3*)	70.9	4.52 (t, 9.6)	71.05	4.79 (t, 9.6)	71.41
							4.37 (t, 9.6)	88.13	4.31 (t, 9.6)	70.58
G3, G3'	3.13 (*m*)	68.53,	4.05 (*t*,*9.3*)	80.0	4.12 (*t*,*9.3*)	79.9	4.31 (t, 9.6)	80.74	4.13 (t, 9.6)	80.20
		68.13					3.94 (t, 9.6)	79.97	4.06 (t, 9.6)	80.30
G4, G4'	2.88 (*m*)	79.73,	4.11 (*t*,*9.3*)	69.7	4.14 (*t*,*9.4*)	69.7	4.35 (t, 9.6)	70.00	4.00 (t, 9.6)	71.19
		79.47					3.90 (t, 9.6)	71.41	4.19 (t, 9.6)	70.01
G5, G5'	3.11 (*m*)	78.48	3.68 (*br d*,*9.3*)	81.6	3.78 (*m*)	79.6	4.02 (bdd, 9.6, 6.0)	80.91	3.87 (ddd, 3.5, 7.0, 9.6)	82.88
							3.88 (bdd, 40.0, 9.6)	81.89	3.68 (dt, 2.0, 2.0, 9.6)	80.99
G6, G6'	3.45 (*m*)	60.05	4.74 (*br d*,*10.8*)	60.0	4.72 (*br d*,*6.7*)	66.6	4.42 (bd, 11.8),	61.62	4.49 (dd, 3.5, 12.0)	62.88
	3.30 (*m*)	59.65	4.39 (*br d*,*10.8*)		4.07 (*br d*,*9.2*)		4.26 (dd, 6.0, 11.8)	63.07	4.12 (dd, 7.0, 12)	61.39
G"1							5.92 (d, 7.6)	38.04	5.88 (d, 7.6)	38.23
G"2							6.21 (dd, 7.6, 4.6)	94.27	5.93 (dd, 7.6, 7.0)	95.92
G"3							4.85 (dd, 4.6, 3.4)	72.74	4.92 (dd, 7.0, 1.2)	72.89
G"4							4.72 (dd, 3.4, 7.2)	73.57	4.63 (dd, 1.2, 8.4)	72.89
G"5							4.57 (ddd, 3.8, 6.3, 7.2)	73.65	4.53 (ddd, 4.0, 6.4, 8.4)	73.25
G"6							4.50 (dd, 3.8, 11.7)	65.03	4.44 (dd, 4.0, 11.6)	65.13
							4.38 (dd, 6.3, 11.7)		4.28 (dd, 6.4, 11.6)	
E1					3.68 (m)	71.9				
E2					4.25 (m)	59.3				
					3.65 (m)					

^a)^ Measured in DMSO; ^b)^ Measured in Pyridine/methanol.

**Table 14:** NMR data for saffloquinoside A, saffloquinoside B, and saffloquioside C.

No.	Saffloquinoside A			Saffloquinoside B				Saffloquinoside C	
	δ_C_^a)^	δ_H_ (mult) ^a)^	HMBC ^c)^	δ_C_^a)^	δ_H_ (mult) ^a)^	δ_H_ (mult) ^b)^	HMBC ^c)^	δ_C_^a)^	δ_H_ (mult)^a)^
1	187.5			196.8				176.9	
2	107.7			112.8				102.5	
3	193.8			188.6				195.7	
4	77.5	6.05 br s (OH)	3,4,5,1"	89.7	5.06 br s (OH)		3,4,5,1"	82.8	
5	173.1			201.7				186.8	
6	116.6			63.6				96.3	
7	179.2	17.42 br s (OH)	2,7,8	182.4	17.83 br s (OH)		1,2,7,8	181.4	
8	117.9	7.48 d (16.0)	7,9,1'	118.3	7.13 d (16.0)	7.11 d (16.0)	7,1'	126.7	7.51 d (15.5)
9	142.4	7.68 d (16.0)	7,1',2',6'	143.6	7.72 d (16.0)	7.71 d (16.0)	7,8,2',6'	135.8	7.16 d (15.5)
1'	126.0			125.9				126.7	
2',6'	130.6	7.54 d (8.0)	4'	131.4	7.60 d (8.5)	7.59 d (8.5)	9,4'	129.0	7.33 d (8.5)
3',5'	116.0	6.83 d (8.0)	1',4'	115.9	6.81 d (8.5)	6.81 d (8.5)	1',4'	115.9	6.72 d (8.5)
4'	160.2	10.11 br s (OH)	3',5'	160.5	10.15 br s (OH)		3',4',5'	159.5	9.76 br s (OH)
1"	83.0	3.51 overlap	5	77.7	4.61 overlap	4.60 d (8.5)	5,3",5"	84.6	3.39 d (9.0)
2"	69.8	3.51 m	1"	69.1	3.32 m			70.1	3.35 t (9.0)
3"	78.1	3.12 m	4",5"	78.3	3.17 m			77.9	3.14 t (9.0)
4"	69.9	2.89 m	3",5	71.0	2.86 dd (9.0, 6.0)		3",5"	69.2	3.05 t (9.0)
5"	81.1	3.02 m	6"	82.0	3.09 m		4"	79.2	2.93 m
6"	61.8	3.63 m	5"	61.9	3.81 m			60.3	3.47 m
		3.31 m			3.44m				
1'''	34.9	3.17 d (15.5)	5,2''',3'''	79.1	3.84 d (10.0)	3.84 d (10.5)	1,5,6	33.8	2.96 d (14.5)
		2.59 d (15.5)							2.35 d (14.5)
2'''	109.5			71.5	3.24 m		3'''	113.9	
3'''	70.2	3.65 m	5'''	78.0	3.07 m		1'''	69.9	3.65 d (9.0)
4'''	69.5	3.71 m	3'''	68.7	3.00 m			68.9	3.76 m
5'''	68.6	3.79 m	6'''	78.1	3.24 m		3'''	69.8	3.78 m
6'''	66.1	3.91m	5'''	60.7	3.55 m 3.38 m		5'''	65.9	3.91 d (11.5)
		3.60 m							3.60 d (11.5)
1''''				125.0					
2'''',6''''				130.8	6.63 d (8.5)	6.63 d (7.5)	3'''',4'''',		
							5''''		
3'''',5''''				114.7	6.46 d (8.5)	6.46 d (7.5)	1'''',2'''',		
							4'''',6''''		
4''''				156.1	9.13 br s (OH)		3'''',4'''',		
							5''''		
7''''				43.5	3.17 d (13.0)		1,5,6,1'''',2'''',6''''		
					3.01 d (13.0)				

^1^H-NMR at 400 MHz; ^13^C-NMR at 100MHz. ^a)^ In DMSO-*d_6_*; ^b)^ In DMSO-*d_6_* + D_2_O; ^c)^ H to C correlations.

**Table 15:** NMR data for HSYA, tinctormin, and SYA.

Position	HSYA		Tinctormin			SYA
	δ_H_^a)^	δ_C_^b)^	δ_H_^a)^	δ_H_^b)^	δ_C_^a)^	δ_C_^a)^
1		189.3 (s)			185.7 (s)	189.4 (s)
2		105.8 (s)			109.2 (s)	106.0 (s)
3		195.0 (s)			195.8 (s)	194.4 (s)
4		85.2 (s)			77.9 (s)	85.8 (s)
5		182.9 (s)			114.6 (s)	183.2 (s)
6		99.3 (s)	6.30 s	6.37 s	101.5 (d)	99.4 (s)
7		179.3 (s)			180.3 (s)	170.0 (s)
8	7.42 d (15.5)	123.1 (d)	7.35 d (16.0)	7.28 d (16.0)	118.9 (d)	123.6 (d)
9	7.31 d (15.5)	135.9 (d)	7.68 d (16.0)	7.63 d (16.0)	140.9 (d)	136.8 (d)
10		127.2 (s)			126.2 (s)	127.8 (s)
11	7.41 d (9.0)	129.2 (d)	7.58 d (8.5)	7.52 d (8.5)	130.4 (d)	130.0 (d)
12	6.77 d (9.0)	115.5 (d)	6.88 d (8.5)	6.81 d (8.5)	115.8 (d)	115.6 (d)
13		158.3 (s)			159.8 (s)	158.6 (s)
1'	3.64 d (9.5)	85.5 (d)	3.30 d (9.5)	3.26 d (9.5)	84.2 (d)	85.8 (d)
2'	3.35 dd (9.5, 4.5)	69.5 (d)	3.45 m	3.37 t (9.5)	69.0 (d)	69.0 (d)
3'	3.11 dd (9.5, 4.5)	78.2 (d)	3.17 m	3.12 t (9.5)	78.3 (d)	79.0 (d)
4'	2.89 td (9.5, 4.5)	69.7 (d)	3.15 m	3.10 t (9.5)	69.2 (d)	70.0 (d)
5'	2.96 td (9.5, 4.5)	80.0 (d)	2.95 m	2.98 dd (9.5, 2)	79.7 (d)	80.7 (d)
6'	3.37 t (10.0)	60.8 (t)	3.50 m	3.41 dd (11, 2)	60.7 (t)	61.2 (t)
	3.60 m		3.54 m	3.52 dd (11, 9.5)		
1"	4.21 d (9.5)	73.8 (d)			140.9 (s)	74.1 (d)
2"	4.12 td (9.5, 4.5)	68.7 (d)			138.2 (s)	71.0 (d)
3"	3.15 dd (10.0, 4.5)	79.1 (d)	4.85 m	4.79 d (3.5)	65.9 (d)	78.0 (d)
4"	3.10 dd (10.0, 4.5)	70.8 (d)	3.57 m	3.46 dd (7.5, 3.5)	73.9 (d)	70.0 (d)
5"	3.05 dd (10, 4.5)	80.5 (d)	3.62 m	3.57 br d (7.5, 3.5)	71.3 (d)	80.7 (d)
6"	3.41 m	61.4 (t)	3.47 m	3.38 dd (11, 3.5)	63.3 (t)	61.7 (t)
			3.67 m	3.58 dd (11, 7.5)		
	3.58 ddd (12, 6.5, 4.5)					
3-OH	18.61 s		17.95 s			
4-OH	4.53 d (4.5)		5.70 br s			
5-OH	9.75 br s					
13-OH	8.30 s		10.07 br s			
2'-OH	4.64 d (4.5)		4.98 d (5.5)			
3'-OH	4.78 d (4.5)		4.94 m			
4'-OH	4.76 d (4.5)		4.81 m			
6'-OH	4.80 t (4.5)		4.11 t (5.5)			
1"-OH			11.26 s			
2"-OH	4.01 d (4.5)					
3"-OH	4.12 m		4.85 m			
4"-OH	4.69 d (4.5)		4.59 br d			
6"-OH	4.45 t (4.5)		4.36 t (4.5)			
-NH-			4.65 d (4.5)			

^1^H-NMR at 400 MHz; ^13^C-NMR at 100 MHz. ^a)^ Measured in DMSO-*d_6_*; ^b)^ Measured in DMSO-*d_6_* + D_2_O.

### 4.6. X-ray Crystallography

**Figure 5:**
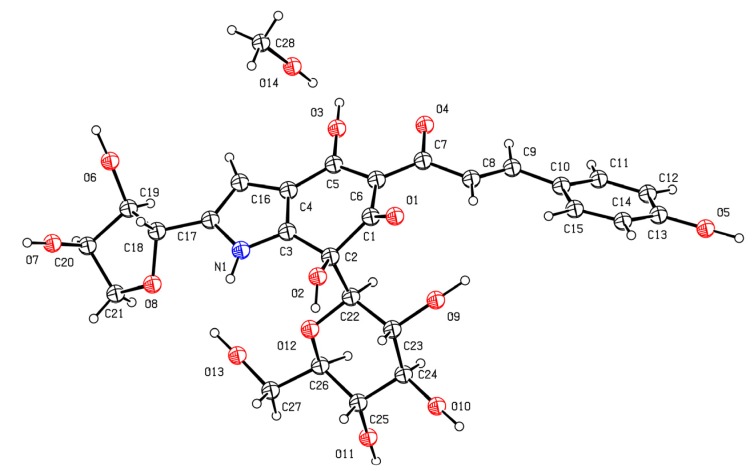
Perspective view of cartormin [86].

In all of the reported literatures on quinochalcone *C*-glycosides, cartormin is the only compound that has been identified by single-crystal X-ray analysis. The original paper includes an image of the solid-state conformation and a molecular ratio of 1:1 for cartormin/CH_3_OH in the crystal state. A view of cartormin was given in Figure 5.

## 5. Biological Activities

Quinochalcone *C*-glycosides exhibit an equally broad spectrum of bioactivities, including modulation of the central nervous system and cardiovascular functions, and their anticoagulative, anti-inflammatory, antioxidant, hepatoprotective, antihypertensive, and anti-tumor activity.

### 5.1. Anticoagulant Effects

HSYA has been shown to have a strong antagonistic effect on platelet activating factor (PAF) receptor. Washed rabbit platelet (WRP) aggregation and rabbit polymorphonuclear leucocytes (PMNs) aggregation induced by PAF were both inhibited by HSYA in a concentration-dependent manner *in vitro* [92]. Subsequently, Liu *et al.* [48] found that HSYA at high dose of 180 mg/kg and at medium dose of 60 mg/kg (90-day daily intraperitoneal injection) induced a prolonged blood coagulation time without influencing the normal blood coagulation process. Meanwhile, the prolonged blood coagulation time was recovered to a normal level on the 28th day after withdrawing HSYA. Besides, SYB could significantly inhibit platelet aggregation *in vitro*, and prolong prothrombin time (PT), thrombin time (TT) and activate partial thromboplastin time (APTT) in a concentration-dependent manner [93]. Therefore, the anticoagulant effects of quinochalcone *C*-glycosides embody the concern of *Carthamus tinctorius* for promoting blood circulation and removing blood stasis.

### 5.2. Effects on Cardiovascular Functions

In developed countries, cardiovascular diseases are the major causes of disability and mortality [94]. Report showed that HSYA could protect Human Umbilical Vein Endothelial Cells (HUVECs) under hypoxia induced injuries by inhibiting cell apoptosis and cell cycle arrest, and increased the Bcl-2/Bax ratio of protein and mRNA, reduced p53 protein expression in cell nucleus [95]. In addition, HSYA could improve cell viability under hypoxia in a concentration-dependent manner by attenuating its cycle arrest and inhibiting its apoptosis, and up-regulate the Bcl-2/Bax ratio, increase vascular endothelial growth factor (VEGF) protein concentration and VEGF mRNA expression and enhance HIF-1α protein accumulation and its transcriptional activity [96]. Besides, Bai *et al.* [59] reported vasodilatation effects of HSYA on pulmonary artery (PA), the results of which suggested that HSYA possessed vascular relaxation effects on rat PA by activating the KV channel in pulmonary vascular smooth muscle cells (PVSMCs). Liu *et al.* [97] revealed that HSYA can provide protection to H9c2 cardiomyocytes against ischemia/reperfusion (I/R) induced apoptosis by up-regulating HO-1 expression through the PI3K/Akt/Nrf2 signaling pathway. Recently, Zhou *et al.* [98] found that the inhibitory effect of HSYA on inflammation was the main mechanism to improve acute myocardial infaration (AMI). Meanwhile, it is reported that HSYA can block the pathogenic effect of AT1-Ab on vascular endothelial and smooth muscle cells [99].

Except for HSYA, Meselhy *et al.* [100] demonstrated that tinctormin exerted a negative inotropic effect which was mediated in part through reduction of the peak Ca^2+^ currents and also due to inhibition of the Na^+^-Ca^2+^ exchange process and/or inhibition of the Ca^2+^ released from the sarcoplasmic reticulum (SR). Wang *et al.* [101] revealed that SYB was able to eliminate the effect of angiotensin II (Ang-II) on vascular endothelial cells (VECs) via regulating [Ca^2+^]_i_, mitochondrial structure and function and inhibiting apoptosis. Current research has shown that SYA can protect cultured rat cardiomyocytes against anoxia/reoxygenation (A/R) via inhibiting cellular oxidative stress and apoptosis [102].

### 5.3. Effects on the Central Nervous System

The second clinical study of HSYA has been approved by the Chinese State Food and Drug Administration (SFDA) for the treatment of brain blood vessel disease. Some reports have shown that HSYA possesses protective and regulatory effects on the central nervous system. HSYA (2, 4, 8 mg/kg, i.v.) could significantly decrease neurological deficit scores and reduce the percentage of infraction in the ipsilateral hemisphere, and also attenuate the elevation of malondialdehyde (MDA) content, the decrease in superoxide dismutase (SOD) activity, and the total antioxidative capacity (T-AOC) in the ipilateral hemisphere and serum, all of which suggested that HSYA might provide neuroprotection against cerebral ischemia/reperfusion injury through its antioxidant action [103]. Fan *et al.* [104] found that treatment of PC12 cells with HSYA can block oxygen-glucose deprivation (ODG) induced apoptosis through suppression of intracellular oxidative stress and mitochondria dependent caspase cascade. Further, Li *et al.* [105] revealed that HSYA could suppress inflammatory responses in BV2 microglia induced by ODG, which was probably associated with the inhibition of the NF-κB signaling pathway and phosphorylation of p38. Furthermore, Ye *et al.* [106] demonstrated that HSYA prevented the injury in cultured cerebral cortical neurons induced by oxygen-glucose deprivation and increased the cell viability by the inhibition of both lactate dehydrogenase(LDH) and NO efflux, and significantly decreased both mRNA and protein levels of IL-1β, TNF-α in ischemic brain tissue. Yang *et al.* [107] revealed that HSYA protected cortical neurons from inhibiting the expression NR2B-containing *N*-methyl-d-aspartic acid (NMDA) receptors and by regulating Bcl-2 family. In addition, Zhu *et al.* [108] found that HSYA could inhibit the opening of mitochondrial permeability transition pore (mtPTP) by a free radical scavenging action in the brain, and this may contribute to the neuroprotective effect of HSYA. They further reported the therapeutic effect of HSYA on focal cerebral ischemic injury, the results suggested that the underlying mechanisms exerted by HSYA might be involved in its inhibitory effects on thrombosis formation and platelet aggregation as well as its beneficial action on the regulation of PGI2/TXA2 and blood rheological changes [109]. Besides, Meanwhile, HSYA treatment inhibited the NF-κB pathway via suppressing proinflammatory cytokine expression and p65 translocation and binding activity while upregulating an anti-inflammatory cytokine. Sun *et al.* [110] suggested that anti-cerebral ischemic mechanism of HSYA may be due to its suppression of thrombin generation and inhibition of thrombin-induced inflammatory responses by reducing Ang-II content. Pan *et al.* [111] investigated the neuroprotection of HSYA against lymphostatic encephalopathy induced brain injury and the associated functional alterations, which was likely regulated by the nitric oxide pathway. Liu *et al.* [112] revealed that HSYA could travel across the blood-brain barrier, significantly reducing the infract volume and improving the neurological functions of rats with ischemia, and lead to relative corrections of the impaired metabolic pathways through energy metabolism disruption, excitatory amino acid toxicity, oxidative stress, and membrane disruption revealed by ^1^H-NMR-based metabonomics.

Parkinson’s disease (PD) is the second most common neurodegenerative disorder, after Alzheimer's disease. Recent studies have found that HSYA can preserve dopamine neuron in integrity and motor function in a model of PD [113].

By whole animal experiments, Wang *et al.* [28] demonstrated that SYB might act as a potential neuroprotective agent against the cerebral ischemia-induced injury in rat brain through reducing lipid peroxides, scavenging free radicals, and improving the energy metabolism. In cellular experiments, they further revealed that SYB protected PC12 cells from H_2_O_2_-induced injury and apoptosis via antioxidant and anti-apoptotic mechanisms [114].

### 5.4. Anti-Inflammatory Properties

Song *et al.* [115] reported the effects of HSYA on lipopolysaccharide induced inflammatory signal transduction in human alveolar epithelial A549 cells, and the results indicated that HSYA suppressed the expression of TLR-4, Myd88, ICAM-1, TNFα, IL-1β and IL-6 at the mRNA and protein level, and inhibited the adhesion of leukocytes to A549 cells. Meantime, HSYA treatment also decreased NF-κB p65 nuclear translocation and inhibited the phosphorylation of p38 mitogen-activated protein kinase (p38 MAPK).

### 5.5. Antioxidant Activity

Atioxidant activity assay *in vitro* by 2,2'-azino-bis(3-ethylbenzthiazoline-6-sulfonic acid) (ABTS) radical cation scavenging showed that the *SC_50_* values of HSYA was 44.39 ± 1.62 μg/mL than vitamin C with *SC_50_* of 4.87 ± 2.22 μg/mL [68]. Besides, carthamin showed significant DPPH, ABTS, hydroxyl, and superoxide free radical scavenging activities, and presented excellent antioxidant ability [116]. In addition, safflomin C, isosafflomin C, methylsafflomin C, and methylisosafflomin C exhibited EC_50_ values similar with those of caffeic acid and ascorbic acid, with Trolox Equivalent Antioxidant Capacity value of 1.34 as determined by ABAP/ABTS assay, and showed strong antioxidant activities against the ABTS radical system [34].

### 5.6. Hepatoprotective Activity

Hepatic fibrosis is a chronic pathological process, and few effective therapies are currently available for treatment of hepatic fibrosis. HSYA, as a nature active ingredient with antioxidant capacity, was able to effectively attenuate oxidative stress mediated injury, thus making it a promising agent for therapy of hepatic fibrosis. Zhang *et al.* [117] firstly reported hepatoprotective effects of HSYA in rats with carbon tetrachloride-induced liver fibrosis. HSYA significantly reduced liver fibrosis, and down-regulated α-smooth muscle actin (SMA), collagen α type I, matrix metalloproteinases (MMP)-9, and tissue inhibitors of metalloproteinases (TIMP)-1 gene expression. This was accompanied by a decreased expression of transforming growth factor (TGF)-β1 and phosphorylation of Smad4. Subsequently, Li *et al.* [118] revealed that HSYA significantly induced apoptosis of hepatic stellate cells (HSCs) in a dose- and time-dependent manner and suppressed the activation of ERK1/2 and ERK1/2-regulated gene expression, including Bcl-2, Cytochrome c, cspase-9, and caspase-3, leading to the enhancement of apoptosis. Recently, it was found that HSYA was able to significantly protect the liver from oxidative stress by stimulating PPARγ activity, reducing cell proliferation and suppressing extracellular matrix (ECM) synthesis [119]. And research has demonstrated that carthamin lowered the serum levels of Alanine aminotransferase (ALT), Aspartate aminotransferase (AST), alkaline phosphatase (ALP) and total protein in liver damage and up-regulated Nrf2, GSTα and NQO1 expressions at the protein level. Meantime, the activities of antioxidant enzymes and level of GSH were elevated by carthamin, while the content of TBARS, an oxidative stress marker, was lessened [116].

### 5.7. Antihypertensive Effects

Nie *et al.* [120] found that HSYA could significantly reduce blood pressure and heart rate, which may be related to activation of BK(Ca) and K(ATP) channels. For patients who responded poorly to the singly-used captopril, Fu *et al.* [121] revealed that either SYA nor captopril could reduce the blood pressure, however, a simultaneous oral administration of the combination of SYA and captopril demonstrated effective blood pressure reduction, suggesting that SYA may synergize the effect of captopril or alter pharmacokinetics of captopril.

### 5.8. Anti-Tumor Activity

Xi *et al.*, brought attention to the notable anti-tumor activity of HSYA. For transplantation tumor of gastric adenocarcinoma cell line BGC-823 in nude mice, they reported that HSYA (0.056 g/L and 0.028 g/L) can inhibit the growth of BGC-823 transplantation tumor, and inhibit tumor angiogenesis by decreasing the mRNA expression of VEGF and bFGF [122]. Subsequently, they revealed that HSYA can induce the apoptosis of tumor cell [123]. Further, it was found that HSYA could inhibit the protein or mRNA expression of MMP-9 in tumor tissue to reduce the degradation of blood vessel membrane, restrain the migration of blood vessel and to decrease the tumor vascularization [124]. Recently, they found that HSYA antagonized tumor angiogenesis may be related to protein expression inhibition of VEGF, and HIF-1α and weakening the phosphorylation of KDR protein and its gene expression to inhibit the activation of endotheliocyte and impede the induction of tumor oxygen-poor microenvironment to angiogenesis [125]. Except for HSYA, Wu *et al.* [126] have reported that carthamin can inhibit the cytoactive and colony formation of K562 leukemic cells and induced K562 leukemic cells to the hemoglobin end cells in a concentration-dependent manner.

### 5.9. Anti-Diabetic Properties

Diabetes is well known to cause vascular complication by activating multiple pathways of biochemical dysfunction. One of these pathways is increased protein glycation by the reactive physiological dicarbonyl compound methylglyoxal (MGO) [127]. Thus, Ni *et al.* [128] investigated the inhibitory effects of HSYA on protein glycation *in vitro*. HSYA concentration dependently decreased advanced glycation end products (AGEs) formation with maximum inhibitory effects at 1 mM by 95%, and also significantly inhibited MGO-medicated protein modification and subsequent cross-linking of proteins. Meanwhile, HSYA exhibited its antiglycation effects on AGEs production with maximum inhibitory effects of HSYA at 1 mM by 84%. They further revealed that HSYA inhibited human brain microvascular endothelial cells (HBMEC) apoptosis and MGO-induced injury by suppressing AGEs accumulation [129].

### 5.10. Other Activities

Kong *et al.* [130] firstly provided preclinical evidence for the protective effect of HSYA against photoaging, which may be related to the anti-oxidative property of HSYA and mediation by promoting endogenous collagen synthesis. Besides, HSYA could exert protective effect against LPS-induced acute lung injury via inhibiting p38 MAPK, NF-κB p65 activation and altering inflammatory cytokine expression [131]. Similarly, Wu *et al.* [132] revealed that HSYA could alleviate early inflammatory response of bleomycin-induced mice lung injury.

## 6. Conclusions

*Carthamus tinctorius* is a valuable plant resource as a medicine and food for dual purposes. It is well known that quinochalcone *C*-glycosides are the major ingredients in the florets of *Carthamus tinctorius*. Recently, quinochalcone *C*-glycosides were found to have multiple pharmacological activities, which has triggered much new research, yet the mechanisms of action of the quinochalcone *C*-glycosides have not been fully elucidated, so the possible physicochemical properties resulting from their chemical features should be taken into consideration when studying their chemical genetics and molecular pharmacology. In addition, solution structures and the different tautomeric forms of the compounds are closely related to their bioactivities [133]. Weeks *et al.* [134] reported that a shift from the keto-enamine to the enolimine tautomer of pyridoxal 5'-phosphate is associated with a loss of activity in cystathionine-synthase. In this review, the structures of the quinochalcone *C*-glycosides in some solvents have been discussed. Due to lack of adequate X-ray analysis, absolute configurations of most quinochalcone *C*-glycosides have not been elucidated. Besides, quinochalcone *C*-glycosides are very unstable, which represent an obstacle during extraction and conservation. Consequently, lots of researchers have been committed to synthesizing quinochalcone *C*-glycosides. However, on the one hand, the *C*-glucopyranosylquinochalcone structure is unique and is present as a complex mixture of keto-enol tautomers. On the other hand, the molecular basis of their biosynthesis pathways has not been elucidated clearly. Both of them provide an obstacle for the total synthesis of quinochalcone *C*-glycosides.

With rapid advances in nanoscience and nanotechnology, nano-materials have been widely applied in mechanics, magnetics, electrics, thermotics, photics, biomedicine, and so on. Hence, the integrated studies of nano materials (e.g., magnetic nanomaterials, including magnetic nanoparticles and nanosensors) and quinochalcone *C*-glycosides will be a hotspot from which new nanomedicines can be invented. Meanwhile, with the improvement of living standard, public concern over the use of synthetic dyes in foods has grown rapidly and the general public prefer the use of natural dyes in foods [63,81]. Water-soluble yellow pigments of quinochalcone *C*-glycosides have strong commercial value as cost-effective valuable colorants which currently are added to juices, yogurt, gelatin desserts, and candy to make more appealing beverages, dairy products, and confectionaries [72,135], but it is important to note that SY has weak clastogenic effects [136]. Therefore, we must follow the principles of benefit-enhancing and harm-avoiding, to the rational development and application of quinochalcone *C*-glycosides.
